# Effects of Charge Density on Spread Hyperbranched Polyelectrolyte/Surfactant Films at the Air/Water Interface

**DOI:** 10.1021/acs.langmuir.3c01514

**Published:** 2023-10-15

**Authors:** Javier Carrascosa-Tejedor, Andrea Tummino, Bence Fehér, Attila Kardos, Marina Efstratiou, Maximilian W. A. Skoda, Philipp Gutfreund, Armando Maestro, M. Jayne Lawrence, Richard A. Campbell, Imre Varga

**Affiliations:** † Division of Pharmacy and Optometry, Faculty of Biology, Medicine and Health, University of Manchester, Oxford Road, Manchester M13 9PT, U.K.; ‡ Institut Laue-Langevin, 71 Avenue des Martyrs, CS20156, Grenoble 38042, France; § CEA Commissariat à l’Energie Atomique et aux Energies Alternatives, 17 Rue des Martyrs, Grenoble Cedex 9 38054, France; ∥ Institute of Chemistry, Eötvös Loránd University, 112, Budapest H-1518, Hungary; ⊥ Department of Chemistry, Faculty of Education, J. Selye University, Komárno 945 01, Slovakia; # ISIS Neutron and Muon Source, Rutherford Appleton Laboratory, Harwell Campus, Didcot OX11 0QX, U.K.; ¶ Basque Foundation for Science, Plaza Euskadi 5, Bilbao 48009, Spain; ∇ Centro de Fısica de Materiales (CSIC, UPV/EHU)—Materials Physics Center MPC, Paseo Manuel de Lardizabal 5, San Sebastián E-20018, Spain

## Abstract

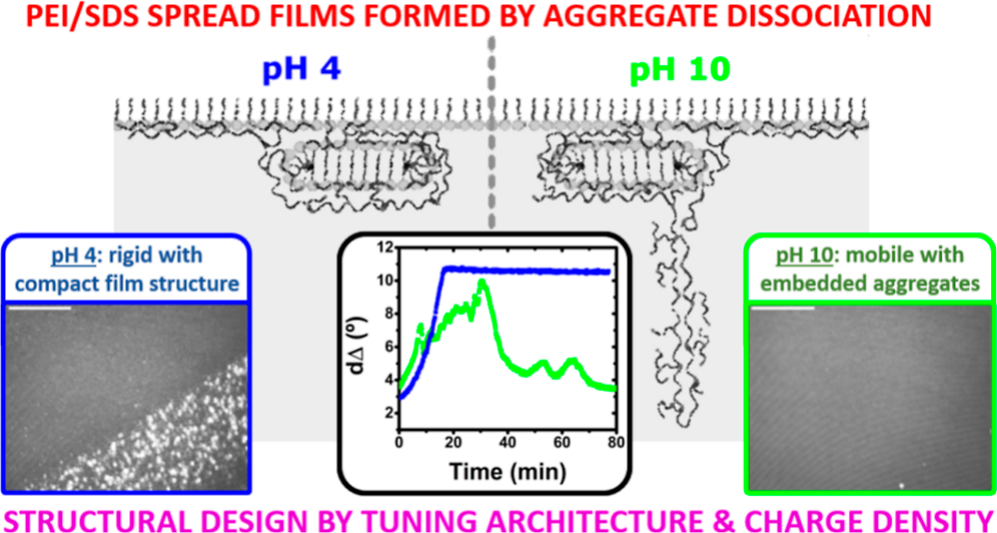

The interfacial structure and morphology of films spread from hyperbranched polyethylene imine/sodium dodecyl sulfate (PEI/SDS) aggregates at the air/water interface have been resolved for the first time with respect to polyelectrolyte charged density. A recently developed method to form efficient films from the dissociation of aggregates using a minimal quantity of materials is exploited as a step forward in enhancing understanding of the film properties with a view to their future use in technological applications. Interfacial techniques that resolve different time and length scales, namely, ellipsometry, Brewster angle microscopy, and neutron reflectometry, are used. Extended structures of both components are formed under a monolayer of the surfactant with bound polyelectrolytes upon film compression on subphases adjusted to pH 4 or 10, corresponding to high and low charge density of the polyelectrolyte, respectively. A rigid film is related to compact conformation of the PEI in the interfacial structure at pH 4, while it is observed that aggregates remain embedded in mobile films at pH 10. The ability to compact surfactants in the monolayer to the same extent as its maximum coverage in the absence of polyelectrolyte is distinct from the behavior observed for spread films involving linear polyelectrolytes, and intriguingly evidence points to the formation of extended structures over the full range of surface pressures. We conclude that the molecular architecture and charge density can be important parameters in controlling the structures and properties of spread polyelectrolyte/surfactant films, which holds relevance to a range of applications, such as those where PEI is used, including CO_2_ capture, electronic devices, and gene transfection.

## Introduction

Polyelectrolyte/surfactant (P/S) mixtures have been well studied both in the bulk^1–4^ and at supported^5–8^ and fluid^9–14^ interfaces. The interest in understanding and developing these materials lies in their widespread use in everyday products, such as detergents,^1^ cosmetics,^15^ pharmaceuticals,^16^ and lubricants.^17^ The inherent nonequilibrium nature of the aggregates formed by P/S mixtures has presented a significant challenge in data interpretations over the years, although progress in this matter has been made over the last 2 decades.^11,13,18^ A key nonequilibrium aspect is related to the phase behavior, as complexes form during mixing that have low charge density, lack colloidal stability, and as a result aggregate.^19^ Complete phase separation may take place over several days, weeks, or even months, depending on the concentration and colloidal stability of the aggregates.^13,20^ When the surfactant is present in a sufficiently large excess, any P/S aggregates formed during mixing can develop kinetic stability due to excess surfactant adsorption on their surface.^19,21,22^ These aggregates typically do not disperse into individual charged P/S complexes if the bulk composition changes and the ionic strength is low.^20,22–24^ This effect manifests even in the mixing of the components when local concentration gradients result in the rapid formation of kinetically trapped aggregates that remain intact even if the overall sample composition is in the equilibrium one-phase region.^25,26^

There are various facets of the ways in which nonequilibrium effects present in P/S mixtures can influence their interfacial behavior. Our initial research focus was to understand depletion effects when aggregation reduces the free surfactant and complex concentrations.^13^ Focus turned later to how the aggregates themselves can influence the interfacial properties when they become embedded in films, such as being transported under gravity.^27^ Work on the mixture poly(ethylene imine)/sodium dodecyl sulfate (PEI/SDS) at the static air/water interface showed that aggregates are embedded in adsorbed layers at pH 10 but not pH 4.^28^ While a study of the same system at the dynamic air/water interface of an overflowing cylinder showed that there can be even more material delivered to the interface by the dissociation of aggregates and Marangoni spreading of their components than the conventionally assumed diffusion and adsorption of complexes from the bulk.^29^ Marked consequences for the use of polyelectrolytes and surfactants under technologically relevant conditions were implied by the findings.

A few years ago, using the poly(sodium styrenesulfonate)/dodecyltrimethylammonium bromide (NaPSS/DTAB) system, we developed a new methodology to form highly efficient spread P/S films by dropping onto the surface of pure water a small aliquot of neutral aggregates.^30^ The dissociation of the aggregates accompanied by the Marangoni spreading of their components results in the formation of a film that exhibits a further nonequilibrium effect by remaining trapped at the interface due to the entropy associated with counterion release into the bulk solution. A much higher surface excess results in the spread films than from the adsorption of complexes from the equivalent concentrations of the same components from the bulk, and no organic spreading solvent is required. Together, these points hint at potential cost and environmental improvements in the use of these films for potential transfer applications.^31–33^

We went on to explore effects of aggregate charge and ionic strength of the subphase, again using the NaPSS/DTAB system.^34^ It was shown that overcharged aggregates (with a surfactant excess on their surface) result in spread films that upon either successive spreading or compression of the surface area exhibit extended structures (ESs) of additional material beneath the “surface monolayer”, which itself is defined as a layer of surfactant in contact with air and polyelectrolytes bound to the headgroups. It was also shown that increased ionic strength of the subphase switched off ES formation as a result of enhanced film equilibration with the bulk.^34^ Recent works turned to exploiting polypeptide/surfactant interactions, where the stratified layer structure of ESs in poly l-lysine/sodium dodecyl sulfate (PLL/SDS) spread films were resolved with respect to the surface area,^35^ and a comparison of the behavior of polypeptide/surfactant spread films that exploit different specific interactions has been delivered.^36^ Three-dimensional control of the film structure through reversible tuning of the coverage not only of the surface monolayer but also of the ESs was robustly demonstrated in these studies.

Still relatively little work has been carried out on spread films that exploit the aggregate dissociation mechanism. Such films have been investigated for four systems involving charged linear polyelectrolytes,^13,30,34–36^ but there is no work involving hyperbranched polyelectrolytes or ones where the charge density can be tuned. As it could be very interesting to exploit the differences in the molecular architecture and/or pH-responsive behavior,^37–39^ the present work focuses on films spread from overcharged hyperbranched PEI/SDS aggregates on pure water subphases adjusted to pH 4 and pH 10. Hyperbranched PEI has technological interest as a result of its statistical ∼1:2:1 distribution of primary, secondary, and tertiary amines with applications in CO_2_ capture,^40^ low-work function modifiers in electronic devices,^41^ and gene transfection.^42^ Overcharged aggregates were chosen simply because they have been shown to result in the formation of ESs for the NaPSS/DTAB^34^ and PLL/SDS^35^ systems. Subphase pH values of 4 and 10 were chosen, as in other studies,^19,29,43^ because PEI is highly cationic in the former case and barely charged in the latter, and the values combined with the 750 kDa molecular weight, matched conditions used in previous studies on PEI/SDS mixtures conducted under static^28^ and dynamic^29^ conditions.

The aim of the present work is to understand the effects of charge density on hyperbranched PEI/SDS films spread using the aggregate dissociation mechanism with respect to film compression. It is the first study of its kind on hyperbranched P/S spread films and represents a key step in the development of the new film formation methodology capable of producing highly efficient films with controllable structures for potential applications. A key objective is to resolve differences in the structure and behavior of spread hyperbranched PEI/SDS films with the spread linear PLL/SDS films studied previously. Following characterization of the charge of the bulk complexes to select the composition of mixtures used to form the aggregates for film preparation, surface-sensitive techniques are applied to the films under static and dynamic conditions including surface pressure–area (Π–*A*) isotherms, ellipsometry, Brewster angle microscopy (BAM), and two different implementations of neutron reflectometry (NR).

## Materials and Methods

### Materials

750 kDa hyperbranched PEI solution (50% in water), SDS, *d*_25_-SDS (*d*-SDS) used for the low-*Q*_*z*_ implementation of NR, ethanol (≥99.8%), D_2_O, NaOH, and HCl were purchased from Sigma-Aldrich, while *d*-SDS used for the full-*Q*_*z*_ implementation of NR was supplied by the ISIS Deuteration Facility. PEI and *d*-SDS from Sigma-Aldrich and all solvents were used as received. SDS and *d*-SDS from the ISIS Deuteration Facility were recrystallized twice in ethanol followed by drying under vacuum. Ultrapure water was generated by passing deionized water through a Milli-Q unit (total organic content ≤4 ppb and resistivity = 18 MΩ·cm).

Note that SDS is prone to slow hydrolysis in acidic and basic media to produce dodecanol as an impurity.^44^ Trace dodecanol is highly surface active in SDS solutions due to its greater driving force for adsorption. This difference in the driving force is diminished, however, for oppositely charged P/S films, as they form electroneutral surface monolayers.^30,34,35^ Validation of our approach includes the following: (1) all SDS and *d*-SDS solutions were freshly made, (2) Π–*A* isotherms of PEI/SDS films recorded with recrystallized SDS and non-recrystallized *d*-SDS exhibit qualitatively similar features (see Section S1 of the Supporting Information), (3) data from the low-*Q*_*z*_ implementation of NR presented below exhibit no indication of gradual adsorption of dodecanol over successive compression/expansion cycles, and (4) data from ellipsometry presented below exhibit no indication of gradual dodecanol adsorption over time.

### Sample Preparation

A stock solution of 2000 ppm PEI was prepared by diluting the original PEI solution as supplied in water and rotating the vial for a few hours. This stock was further diluted in water to 200 ppm. Stock solutions of SDS 10 mM and *d*-SDS were prepared and diluted to the required concentration for each experiment. It is worth noting that 100 ppm PEI solutions have a pH of 10.^19^

Fresh mixtures of PEI/SDS aggregates were prepared by rapidly mixing, i.e., pouring together quickly, the same volumes of the polyelectrolyte and surfactant solutions at double their final concentrations. The mixtures were extracted within 3–5 s after mixing the components and spread within the following 60 s to limit the growth of large aggregates prior to film formation. Because this study focuses on the influence of subphase pH on the dissociation of PEI/SDS aggregates and the spread film properties, only the pH of the subphase was adjusted to 10 (low PEI charge density) or 4 (high PEI charge density) using concentrated solutions of NaOH or HCl, respectively, rather than the pH of the mixed PEI/SDS spreading solutions.

### Electrophoretic Mobility

Measurements of the electrophoretic mobility, μ, were recorded using a Zetasizer Nano ZS90 and the M3-PALS technique (Malvern Instruments Ltd., U.K.) to determine the electrophoretic mobility of the aggregates.^45^ In this case, a 10,000 ppm stock solution was filtered using a 0.2 μm membrane and the polymer content was determined by measuring the dry mass (following drying in an oven at 110 °C) of 10 g of the stock solution. Based on the concentration obtained, 200 and 1000 ppm stock solutions were prepared. Measurements were performed at a constant concentration of PEI (100 or 500 ppm) as a function of the SDS concentration. A range of SDS concentrations was used to characterize the aggregates from an excess of PEI to an excess of SDS. Five parallel electrophoretic mobility measurements were recorded at each surfactant concentration and the values were averaged. For mixtures containing 100 ppm PEI, an SDS concentration of 2.5 mM was chosen to create overcharged aggregates (see Section S2 of the Supporting Information).

### UV–vis Spectroscopy

UV–vis spectroscopy data were recorded to evidence the weak scattering of samples much below the composition of charge neutrality for samples at pH 10. The optical density (O. D.) can be expressed as
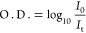
1where *I*_0_ is the incoming light intensity and *I*_t_ is the transmitted light intensity. The measurements were performed using a PerkinElmer Lambda 2 UV–vis spectrophotometer in a quartz cell that had a path length of 1 cm. The optical density at 400 nm was analyzed, as neither PEI nor SDS has any absorption band at wavelengths longer than 350 nm. Measurements were carried out 1 min after mixing the components.

### Langmuir Technique

A Langmuir trough allows the study of the dynamic response of films at the air/water interface during compression/expansion cycles,^46^ as well as evaluation of their stability when set to a given compression ratio, defined as *A*_0_/*A*, where *A* is the trough area and *A*_0_ is the maximum trough area on which the film was spread.^47^ Values of Π shown are the difference between the surface tension of pure water and that of the measured film. Different troughs were used in this work depending on the setup needed to combine with the techniques described below. All the troughs were equipped with two barriers that moved symmetrically. The surface pressure was recorded using the Wilhelmy plate method. A Kibron G1 trough (Finland, *A*_max_ = 166.4 cm^2^) was used to record the Π–*A* isotherms of PEI/SDS films during successive compression/expansion cycles. Other troughs used in combination with reflectometry techniques are detailed below. Prior to the experiment, the trough was carefully cleaned with detergent, ethanol, and Milli-Q water. An aliquot of 290 μL of a fresh mixture of PEI/SDS was then spread on the subphase at the maximum surface area of the trough. After 10 min of equilibration, the films were compressed by a factor of 2 at a rate of 4.5 cm^2^/min. The spread volumes and maximum compression ratio were scaled from one experiment to the other considering the dimensions of the troughs used.

### Ellipsometry

Ellipsometry is a highly sensitive and precise optical technique used to determine changes in the local surface excess of material at the air/water interface.^48^ It is based on the changes in the polarization of light upon reflection at an interface. These changes are defined by the ellipticity, ρ, which is related to the experimentally determined ellipsometric angles Ψ (amplitude change) and Δ (phase shift) by the following equation
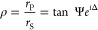
2where *r*_P_ and *r*_S_ are the reflectivity coefficients of the parallel and perpendicular components of the electric field, respectively. When ellipsometry is applied in the study of thin films at the air/water interface, often only values of Δ are considered because Ψ is practically insensitive to changes in the surface excess,^49^ so only Δ values are presented. The ellipsometry data is presented as dΔ = Δ_P/S_ (for the P/S film) – Δ_water_ (for pH-adjusted pure water).^50^

Ellipsometry was used to examine the stability of PEI/SDS films. In this case, spread films were prepared, and then the trough area was reduced until Π = 40 mN/m was achieved, after which Π was held constant. An aliquot of 2000 μL PEI/SDS aggregates was spread in this case to ensure that Π = 40 mN/m was reached before reaching the minimum trough area. The ellipsometry data were recorded using an Accurion EP4 ellipsometer (Germany) equipped with a blue diode laser with a wavelength of λ = 489.2 nm coupled to a Kibron G2 trough (Finland, *A*_max_ = 280 cm^2^). An angle of incidence of 50° and a data acquisition rate of 0.1 Hz were used. Because of the fast acquisition rate and the relatively small probed area (∼1 mm^2^), ellipsometry can detect the presence of inhomogeneities on the μm scale as temporal fluctuations in the signal.^49,51^

### BAM

BAM experiments were performed to image the PEI/SDS films during the acquisition of the Π–*A* isotherms to observe the in-plane organization of the films and the presence of inhomogeneities on the μm scale.^52^ An Accurion Nanofilm EP3 Brewster angle microscope (Germany) equipped with a 50 mW Nd/YAG laser (λ = 532 nm), a CCD detector, and a 10× magnification objective was used. BAM images were taken at the Brewster angle of water (53.1°) without background subtraction. The presence of a PEI/SDS monolayer causes a change in the refractive index of the interface, which results in the reflection of light, resulting in a homogeneous gray image, yet the presence of ESs beneath the surface monolayer appears as white bands or discrete regions.

### NR

NR is a powerful technique used to resolve the composition and structure of films at the air/water interface.^53^ A typical NR experiment consists of shining a neutron beam on an interface at grazing incident angles and recording the neutron reflectivity, *R*, which is the ratio between the intensity of the reflected and the incident neutrons, recorded as a function of the momentum transfer normal to the interface, *Q*_*z*_, defined as
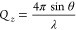
3where λ is the wavelength of the neutron beam and θ the angle of incidence. In this work, specular NR has been used, i.e., the angle of incidence and reflection are equal. Isotopic contrast variation is exploited to vary the scattering length density (SLD or ρ) of a material, defined as the sum of the scattering length, *b*, of each atom divided by the molecular volume, *V*_m_. The values of *b*_*i*_, *V*_m_, and ρ of the materials used in this work are listed in Table 1; values of *b*_*i*_ of PEI have been calculated considering a 90% proton/deuterium exchange as resolved by small-angle neutron scattering measurements,^54^ noting that the protonation state of PEI depends on its surfactant binding, and bulk binding estimates of 60% at pH 4 and 20% at pH 10 are discussed in Section S2 of the Supporting Information.

**Table 1:** Scattering Length (*b*_*i*_), Molecular Volume (*V*_m_), and Scattering Length Density (ρ) Used in This Work for the Different Components Studied

component	*b* (fm)	*V*_m_ (Å^3^)	ρ (× 10^–^^6^ Å^–^^2^)
SS headgroups	29.71	61	4.87
C_12_H_25_-chains	–13.76	352	–0.39
C_12_D_25_-chains	246.53	352	7.00
SDS molecules	15.95	413	0.39
d_25_-SDS molecules	276.24	413	6.69
PEI (in ACMW) pH 4	3.21	78	0.41
PEI (in D_2_O) pH 4	16.7	78	2.15
PEI (in ACMW) pH 10	4.33	78	0.56
PEI (in D_2_O) pH 10	14.45	78	1.86

First, the low-*Q*_*z*_ implementation of NR was used to determine changes in the composition of PEI/SDS films during successive compression/expansion cycles. A detailed description of the physical basis of this approach has been reported elsewhere.^30,34^ In short, two independent measurements were recorded on the minute time scale involving PEI with SDS or *d*-SDS on ACMW (air contrast matched water; a mixture of 8.1% v/v D_2_O in H_2_O). The Π–*A* isotherm experiment was reproduced using a Nima trough (UK, *A*_max_ = 265 cm^2^). Measurements were performed on the FIGARO reflectometer at the Institut Laue–Langevin (Grenoble, France) using a wavelength range λ = 2–16 Å, 10% resolution in d*Q*_*z*_/*Q*_*z*_ and an angle of incidence θ = 0.62°. The composition of the P/S films were resolved in terms of the surface excess of each component *i*, Γ_*i*_, defined as
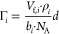
4where *V*_f,*i*_ is the volume fraction of component *i*, *N*_A_ is the Avogadro’s number, and *d* is the film thickness. The two data sets can be used to determine Γ_PEI_ and Γ_SDS_ by solving
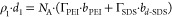
5
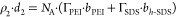
6where ρ_1_ = 4 × 10^–6^ Å^–2^ and ρ_2_ = 1 × 10^–6^ Å^–2^ are the SLD values used in the model fits and *d* is the fitted thickness. As data using only low-*Q*_*z*_ values (0.01–0.03 Å^–1^) were used, these measurements are insensitive to the film structure. The high scattering length of *d*-SDS enables precise determination of Γ_SDS_ excess using 1 min slices. However, as PEI has a much lower scattering length and is not available in its deuterated form, the determination of Γ_PEI_ requires 2 min slices to reduce the uncertainty. The background was not subtracted from the data, and values of 1.36 × 10^–5^ and 1.34 × 10^–5^ were applied in the model using 1 min and 2 min slices, respectively. Motofit^55^ was used to batch fit the thickness of a single layer model with an arbitrary roughness of 4 Å; fitting at low-*Q*_*z*_ is not sensitive to the roughness.

Second, the full-*Q*_*z*_ implementation of NR was used to determine the structure normal to the interface of PEI/SDS films. For that purpose, the ellipsometry experiment, where samples were held at a constant surface pressure of 40 mN/m, was reproduced using a Nima trough (UK, *A*_max_ = 700 cm^2^). Measurements were performed on the INTER reflectometer at the ISIS Neutron and Muon Source (Didcot, UK) using a wavelength range λ = 1.5–16 Å, 5.5% resolution in d*Q*_*z*_/*Q*_*z*_, and two angles of incidence (θ_1_ = 0.8° and θ_2_ = 2.3°). Three different isotopic contrasts were recorded: *d*-SDS/ACMW, *d*-SDS/D_2_O, and SDS/D_2_O. D_2_O was used to calibrate the absolute reflectivity. Co-refinement of data from the three different contrasts was carried out in Motofit^55^ using a model with up to 6 stratified layers, as detailed in Table 2.

**Table 2:** Stratified Layer Structure of PEI/SDS Films Indicating the Composition of Each Layer

	layer	composition
surface monolayer	1	SDS tails
	2	SDS headgroups + PEI + solvent
	3	PEI + solvent
ESs	4	SDS bilayer + solvent
	5	PEI + solvent
	6	PEI + solvent (pH 10 only)

There are four parameters used to define each layer: the thickness (*d*), SLD, solvent volume fraction (*V*_f,solvent,*j*_), and roughness of the upper interface. Given the large number of fitting parameters associated with the model in different isotopic contrasts, to limit the number of free fitting parameters, parameters for layers 2 and 6 were modeled rather than fitted. Three physical constraints were applied. First, the surface excess of surfactant chains (in layer 1) and headgroups (in layer 2) are the same. Second, as other spread P/S films present a 1:1 molar stoichiometry,^30,34,35^ stoichiometric charge binding (taking into account the calculated charge density of PEI estimated above) is present around the headgroups (layer 2). Third, consistent with the presence of a higher amount of PEI needed to match the P/S stoichiometry resolved in the low-*Q*_*z*_ analysis than can be accommodated in the first 5 layers of the model, 5% coverage of 100 Å PEI loops was added at pH 10 only (layer 6), which is consistent with the physical picture of the same polyelectrolyte bound to a solid support.^56^ For all layers, the SLD values were fixed and those containing PEI were made consistent with the proton exchange discussed above. The roughness of the upper interface of all layers was fixed at 4.2 Å, which is consistent with the presence of capillary waves considering the surface tension of the system (∼40 mN/m).^57,58^ The backing roughness was also fixed at 4.2 Å except where the diffuse polymer loops (layer 6) was required, for which the value was fixed at 30 Å. Residual background values of 7 × 10^–6^ were used for measurements in ACMW and 2 × 10^–6^ for measurements in D_2_O.

The free fitting parameters to minimize χ^2^ of the global fit are *d* of layers 3, 4, and 5 (each constrained to be the same in all contrasts; also, to avoid an unphysically high density of PEI beneath the surfactant in the ESs, the thicknesses of PEI above and below the surfactant in the ESs (layers 3 and 5) was constrained to be equal), and *V*_f,solvent_ of layers 3, 4, and 5 (constrained to be the same in all contrasts for layer 3 but only in the *d*-SDS/ACMW and SDS/D_2_O contrasts for layers 4 and 5, as the data from the *d*-SDS/D_2_O contrast did not support the presence of ESs). It may be noted that the necessity to vary the volume fraction of the ESs according to the isotopic contrast matches the approach and findings in two recent studies involving P/S adsorbed layers that also exhibit ESs.^59,60^ The values of the parameters fitted were restricted using the genetic algorithm as follows: 2–15 Å for *d* of layers 3 and 5, 20–30 Å for *d* of layer 4, and 0–1 for *V*_f,solvent_ of layers 3–5. The global fit described was performed iteratively at different values of the thickness of layer 1, which was fixed at its optimal value for the final global fit of the free fitting parameters in layers 3–5 noted. The uncertainty of each parameter has been calculated as the difference between the optimized parameter and the value of that parameter that results in a 10% increase in χ^2^.

## Results and Discussion

Prior to presentation and discussion of new results on hyperbranched spread PEI/SDS films, a brief summary of the general behavior of spread linear NaPSS/DTAB^30,34^ and PLL/SDS^35^ films when manipulated on a Langmuir trough is given. Upon compression of the surface area, Π increases until it reaches a critical value for each system with a discontinuity in the data at ∼28 mN/m. Further compression results in a pronounced plateau in Π. This coincides with the formation of discrete ESs bound to the surface monolayer that increase in coverage with further compression. Reincorporation of material from the ESs back into the surface monolayer occurs upon expansion of the surface area, albeit with a kinetic barrier, showing that the structural changes are reversible.

**Figure 1:**
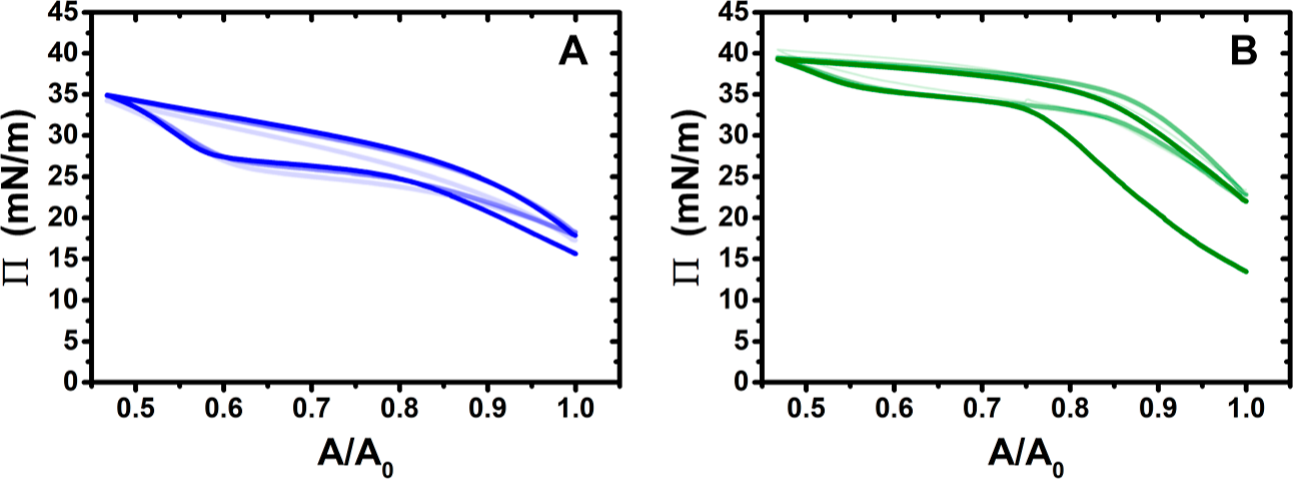
Π–*A* isotherms of PEI/SDS spread films on pure water subphases adjusted to (A) pH 4 and (B) pH 10 during three compression/expansion cycles on a Langmuir trough, where the maximum compression ratio—inverse of the *A*/*A*_0_ scale—is slightly above 2 and successive cycles are darker in color.

Figure 1 shows three compression/expansion cycles of spread PEI/SDS films on a Langmuir trough. The surface pressure upon spreading (maximum *A*/*A*_0_) is around 17 mN/m on a pH 4 subphase and 23 mN/m on a pH 10 subphase. As equivalent aliquots of P/S aggregates were spread on each subphase, this result hints at different efficiencies of the Marangoni spreading process on a subsecond time scale or different interfacial structures formed in the spread film, which is a point we will return to in a later discussion of data from complementary techniques.

The surface pressure at the maximum compression ratio reached (minimum *A*/*A*_0_) of spread films on a pH 4 subphase (panel A) is around 35 mN/m, while that of films spread on pH 10 subphase (panel B) is 40 mN/m. Like the behavior of PLL/SDS spread films,^35^ the successive compression/expansion cycles are similar in appearance, indicating general recovery of material to its existing structures during the expansion, but shifts of the isotherms indicate changes in the morphology of the films upon successive cycles. On the other hand, the general shapes of the isotherms of spread PEI/SDS films are very different to that of NaPSS/DTAB and spread PLL/SDS films. There is no discontinuity at the start of a pronounced Π plateau^34,35^ and instead for the spread PEI/SDS films on both subphases at pH 4 and pH 10, there is a much broader plateau with Π values extending well above 28 mN/m. It may be noted that the surface tension of a layer of PEI/SDS complexes is 30 mN/m (i.e., a surface pressure of ∼43 mN/m),^28^ which hints that a surface pressure of >40 mN/m may be required to saturate the surface monolayer of the spread film upon further compression. The different shapes of the isotherms for spread hyperbranched PEI/SDS films may, therefore, be related to the influence of the macromolecular architectures on the interfacial structures formed in the spread films.

**Figure 2:**
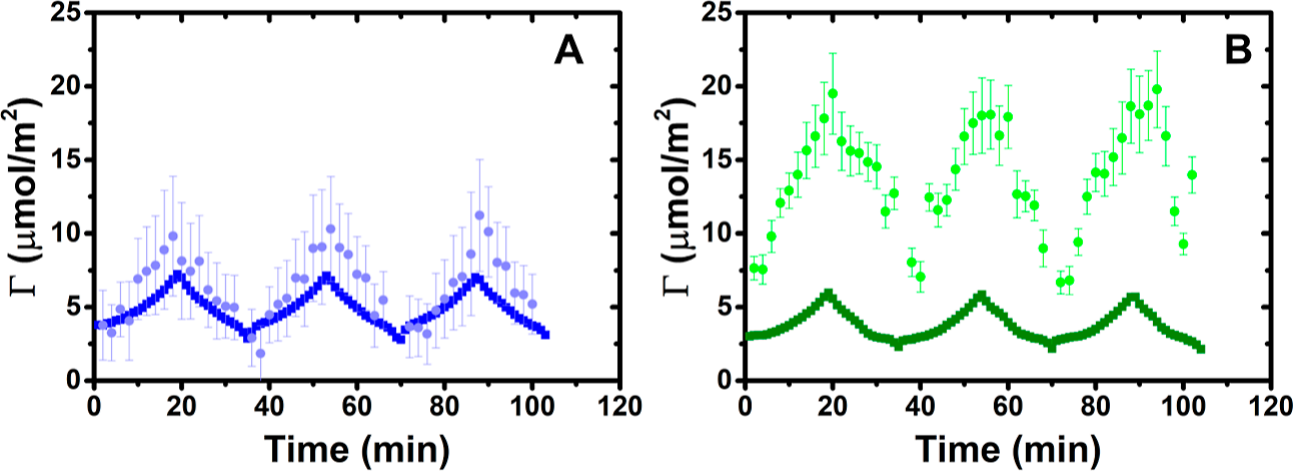
Surface excesses of PEI (lighter circles) and SDS (darker squares) of spread PEI/SDS films on pure water subphases adjusted to (A) pH 4 and (B) pH 10 during three compression/expansion cycles on a Langmuir trough, where the maximum compression ratio is slightly above 2, resolved using the low-*Q*_*z*_ implementation of NR. It may be noted that outliers in the PEI surface excess were deleted for clarity, and the relatively high uncertainties in the PEI surface excess values are related to its low scattering length.

We go on to resolve the film composition in terms of the surface excesses of PEI and SDS, Γ_PEI_ and Γ_SDS_, respectively, also over the three compression/expansion cycles, again at subphase pH values of 4 and 10, using the low-*Q*_*z*_ implementation of NR. For this purpose, two measurements were carried out using the *d*-SDS/ACMW and SDS/ACMW contrasts, from which Γ_PEI_ and Γ_SDS_ are calculated. Figure 2 shows the measured values of Γ_PEI_ and Γ_SDS_ over the three cycles.

The most striking observation is that Γ_SDS_ reaches 7.2 ± 0.1 μmol/m^2^ at pH 4 and 5.9 ± 0.1 μmol/m^2^ at pH 10 by the end of the first compression, which far exceeds the surface excess of SDS at limiting surface coverage at above its critical micelle concentration of 4.2 ± 0.1 μmol/m^2^.^61^ It may be noted that the onset of ES formation for PLL/SDS films occurs upon compression once the surfactant coverage in the surface monolayer reaches 4.0 ± 0.1 μmol/m^2^,^35^ a slightly lower value than the limiting surface coverage of the surfactant alone. Hence, these data strongly imply that, similarly to the films spread from overcharged aggregates for NaPSS/DTAB^34^ and PLL/SDS,^35^ ESs including SDS present beneath the surface monolayer form upon the compression of spread PEI/SDS films on subphases of both pH values studied. As there is no pronounced plateau in the surface pressure isotherms though, it is unclear at what point the onset of ES formation occurs. Indeed, upon spreading, the higher surface pressure yet lower surfactant surface excess at pH 10 compared with pH 4 may be considered at the first paradoxical. However, this may be related to different interfacial structures and proportions of SDS in the surface monolayer versus ESs.

The film stoichiometry, Γ_PEI_/Γ_SDS_, is approximately constant over each whole cycle, suggesting that electrostatic interactions are the primary driver for the binding ratio in the films. Average values of Γ_PEI_/Γ_SDS_ = 1.2 and 3.5 are exhibited at pH 4 and 10, respectively. These differences can be rationalized considering the dependence on the pH of surfactant binding. At pH 4 in the bulk, we have estimated from the binding isotherm noted above (with reference to Section S2 of the Supporting Information) that approximately 60% of the amine groups are protonated, allowing for interactions with the surfactant headgroups in the monolayer, and at pH 10 in the bulk, only 20% of the PEI units are protonated, resulting in a higher amount of PEI required to neutralize the charges of the headgroups. From the Γ_PEI_/Γ_SDS_ values resolved here, it may be the case that the charge of the polyelectrolyte while confined in the film is higher as the corresponding numbers of protonated amine groups at the two respective pH values are 80 and 30%.

**Figure 3:**
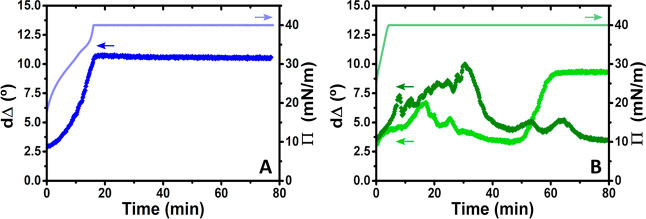
Ellipsometry dΔ (points) and Π (lines) data of spread PEI/SDS films on pure water subphases adjusted to (A) pH 4 and (B) pH 10, where the films are initially compressed to a surface pressure of 40 mN/m and then held at that surface pressure. In panel (B), ellipsometry data from two experiments are shown to highlight the variable nature of the temporal fluctuations.

We examine next the stability of spread PEI/SDS films at a compression ratio of 2.00, at which we have inferred above that ESs have formed, with measurements of dΔ from ellipsometry and surface pressure on a Langmuir trough shown in Figure 3.

With a pH 4 subphase, the values of *d*Δ increase upon surface area compression consistent with the increase in surface excess of both components described above. Once maximum compression is reached, dΔ remains virtually constant with a relaxation of just 3%, suggesting that the ESs formed during compression are very stable with time and that adsorption of trace dodecanol is not significant. There are no temporal fluctuations observed in the data at constant surface pressure, which can be interpreted either in terms of a lack of aggregates embedded in the films, many small aggregates whose average number is practically constant in the area probed by the laser beam, or a higher film stiffness where the signal from any aggregates in the area probed by the laser is constant with time.

With a pH 10 subphase, the maximum surface pressure is achieved with less compression, which is consistent with the higher starting surface pressure of the spread films (25 mN/m as opposed to 18 mN/m). A key difference is observed in the ellipsometry data where there are pronounced temporal fluctuations, and data shown from two equivalent experiments reveal that the fluctuations, within the same bounds of around 3–8°, are seemingly random with time. It follows in this case that the spread films are not rigid and either the number of embedded aggregates or number of ESs fluctuates significantly as they are being transported in and out of the area probed by the laser beam on the second time scale.

**Figure 4:**
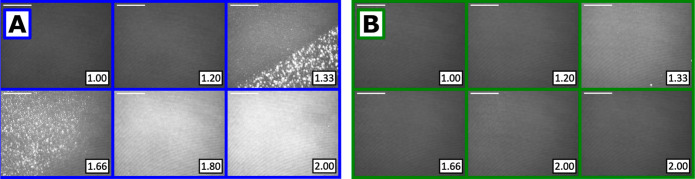
BAM images of spread PEI/SDS films on pure water subphases adjusted to (A) pH 4 and (B) pH 10 during compression of the surface area on a Langmuir trough, where the insets show the values of the compression ratio, *A*_0_*/A*, and the scale bars are 100 μm.

We turn now to optical imaging from BAM, and in this case, prior to a description of the results, it is important to highlight what we may hope to deduce from the data. The technique has a resolution on the μm scale while freshly formed P/S aggregates have a size on the hundreds of nm scale.^30^ Therefore, we would not expect to resolve the presence of individual aggregates embedded in the films, yet we may hope to resolve any lateral association of the aggregates or the presence of ESs. Also, while in the absence of BAM imaging, we do not know the dimensions of the ESs for spread PEI/SDS films, it was possible to resolve a network of ESs in spread PLL/SDS films with a morphology on a length scale of several μm.^35^ Finally, the exposure time of the BAM images was typically 1 s. As a result, whether the intensity fluctuates for images taken at the same compression ratio may reveal information about the mobility or rigidity of the film. Figure 4 shows images recorded with during increase of the compression ratio for spread PEI/SDS films on subphases of both pH values.

With a pH 4 subphase, upon film compression, discrete bright patches on the tens of μm scale are observed from compression ratios of 1.33, yet a longer range order on a length scale of hundreds of μm can also be seen. Upon further compression of the film, the morphology becomes more homogeneous and rigid, and when a compression ratio of 1.80 is reached there is no observed movement in the film. While it would be intuitive to infer that the bright patches observed are the ESs, objectively, these images are not sufficient to distinguish them from embedded aggregates and the full-*Q*_*z*_ implementation of NR below will be required to do so.

With a pH 10 subphase, the films exhibit increasing intensity up to a compression ratio of 1.33, and fluctuations are observed thereafter even up to a compression ratio of 2.00. Even at constant different compression ratios, the images fluctuated in intensity with time and of the two images shown at this compression ratio, the one taken last has a reduction in brightness of 11%. This result explains the key difference in the ellipsometry data of the films on subphases at the two pH values: the films are rigid on a subphase adjusted to pH 4 yet mobile at pH 10.

**Figure 5:**
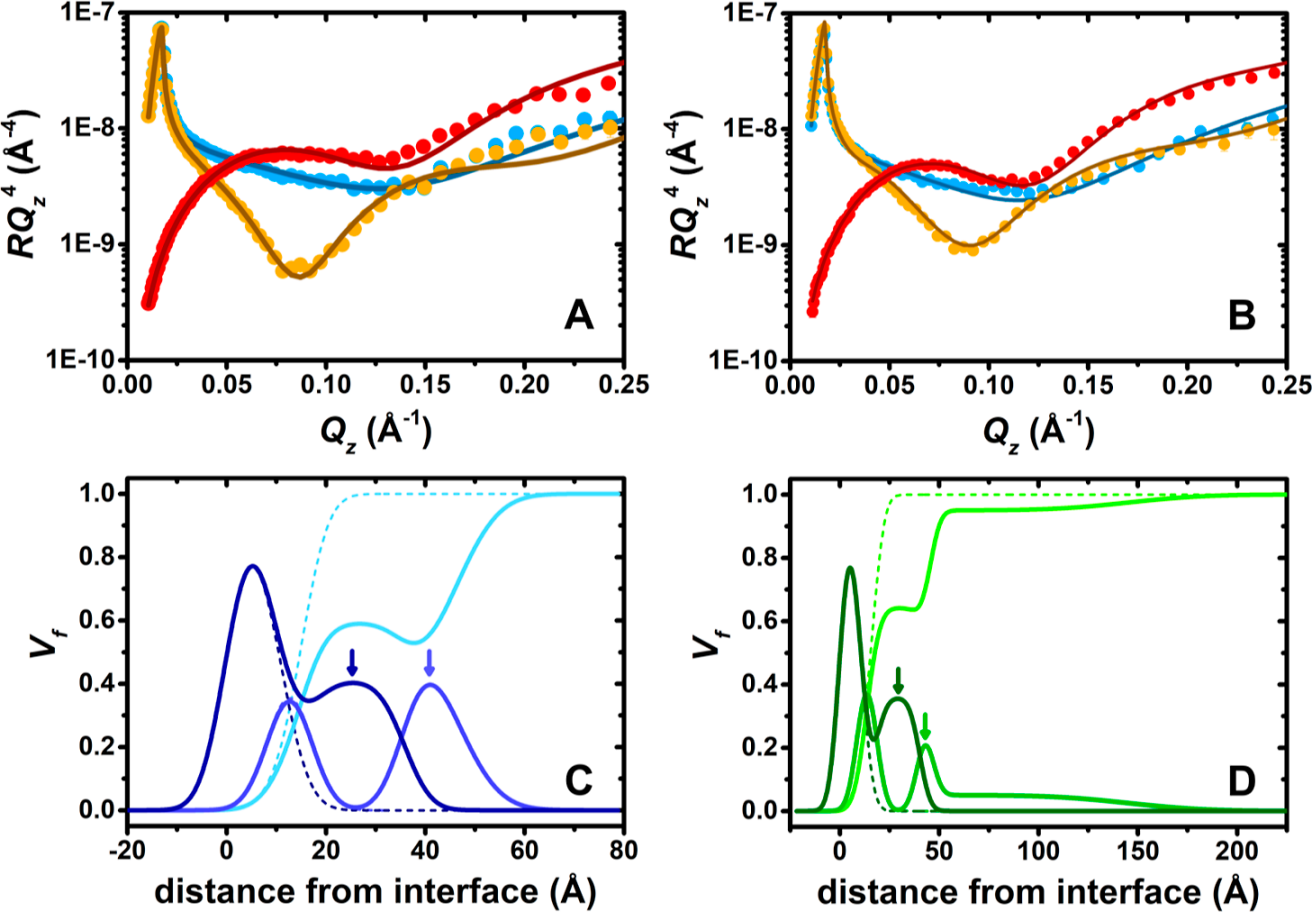
Neutron reflectivity (A,B) data (points) and model fits (lines) of spread PEI/SDS films in three isotopic contrasts involving PEI with *d*-SDS in ACMW (red), *d*-SDS in D_2_O (blue), and SDS in D_2_O (orange) of spread PEI/SDS films on pure water subphases adjusted to (A,C) pH 4 and (B,D) pH 10 and held at 40 mN/m, resolved using the full-*Q*_*z*_ implementation of NR; (C,D) resulting volume fraction profiles for surfactant (dark blue/dark green), polyelectrolyte (blue/green), and solvent (light blue/light green) in the two respective panels. Solid and dashed lines correspond to the volume fraction profiles of the *d*-SDS/ACMW and SDS/D_2_O contrasts presenting ESs and the *d*-SDS/D_2_O contrast presenting only a PEI/SDS monolayer. Arrows in (C,D) indicate the density of SDS in layer 4 and PEI in layer 5 comprising the ESs.

**Figure 6:**
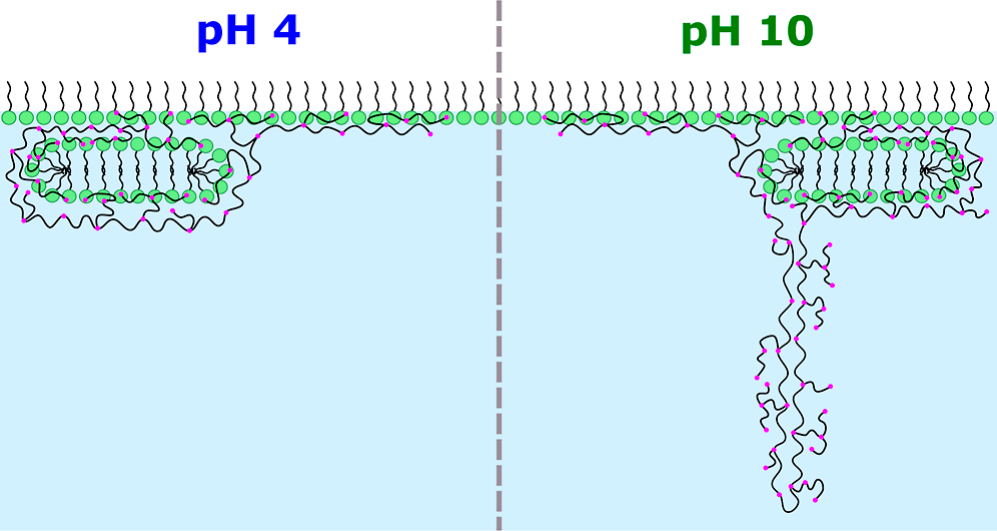
Schematic illustration (not drawn to scale) of the structure of spread PEI/SDS films at 40 mN/m. The hyperbranched structure of the polyelectrolyte is indicated where pink dots indicate nitrogen atoms.

Lastly, the full-*Q*_*z*_ implementation of NR was applied through the acquisition of data recorded in three isotopic contrasts for compressed PEI/SDS spread films. Figure 5 shows data recorded for spread PEI/SDS films compressed and held at 40 mN/m for both studied pH values, as well as the resulting model fits and volume fraction profiles normal to the interface. Here, it may be noted that the ESs in the volume fraction profiles in panels C and D can be seen by additional regions of SDS in layer 4 and of PEI in layer 5 that are indicated with arrows. Also, Table 3 details the thickness and composition of each stratified layer at pH 4 and 10. Further, Figure 6 shows a schematic representation of the general layer structure resolved; its purpose is simply to serve as a helpful visualization, while it is acknowledged that it is not an exact replica due to a lack of resolution of the positions of the branches and backbone in the applied technique.

**Table 3:** Thickness (*d*_*j*_) and Composition Obtained from the Fit of the PEI/SDS Films Spread on a Subphase Adjusted to pH 4 and pH 10, where *j* is the Layer Number, and the Volume Fractions of the ESs (i.e., Layers 4–6) for the *d*-SDS/D_2_O Contrast are Zero, and where Layer 6 Is Required only at pH 10

layer	parameter	pH 4	pH 10
1	*d*_1_ (Å)	9 ± 1	9 ± 1
	composition (*V*_*f*_ %)	100% SDS chains	100% SDS chains
2	*d*_2_ (Å)	4	4
	composition (*V*_*f*_ %)	39% SDS headgroups	39% SDS headgroups
		50% PEI	50% PEI
		11% solvent	11% solvent
3	*d*_3_ (Å)	2 ± 1	5 ± 1
	composition (*V*_f_ %)	80 ± 20% PEI	52 ± 16% PEI
		20 ± 20% solvent	48 ± 16% solvent
4 (*excluding *d*-SDS/D_2_O)	*d*_4_ (Å)	20 ± 1	22 ± 1
	composition (*V*f %)*	41 ± 2% SDS	36 ± 3% SDS
		59 ± 2% solvent	64 ± 3% solvent
5 (*excluding *d*-SDS/D_2_O)	*d*_5_ (Å)	10 ± 1	5 ± 2
	composition (*V*_*f*_ %)*	61 ± 8% PEI	42 ± 16% PEI
		39 ± 8% solvent	58 ± 16% solvent
6 (*excluding *d*-SDS/D_2_O)	*d*_6_ (Å)		100
	composition (*V*_*f*_ %)*		5% PEI
			95% solvent

As noted in the Materials and Methods section, the global fit at each pH value of the data to a stratified layer model involves allowing the volume fraction of layers 4 and 5 (corresponding to the coverage of ESs) to vary according to the isotopic contrast. Indeed, there are no ESs resolved for the contrast involving *d*-SDS/D_2_O while the coverage of these surfactant bilayer (or hemimicelles) varies in the range 36–41% for the two pH values. A fitting demonstration of the need to adopt this approach is presented in Section S3 of the Supporting Information. This issue is attributed to isotope specific effects in the ESs, and it is noted that the varying coverage of ESs has recently been resolved in adsorbed layers of different mixed P/S systems,^59,60^ attributed to varying intermolecular interactions or interactions of molecules with the solvent.^54^ It has also been demonstrated in a separate study that the charge density of PEI can vary from H_2_O to D_2_O depending on the pH.^62^

The thickness of the surfactant chains is 9 ± 1 Å at both pH values. This value corresponds to a surface excess of 4.2 μmol/m^2^, which matches that of a pure SDS monolayer at double its critical micelle concentration,^61^ and it exceeds the value reached for compressed PLL/SDS spread films.^35^ A reason for this slightly higher amount may be the high surface pressure of this measurement of Π = 40 mN/m, which slightly exceeds the maximum surface pressure of Π = 38 mN/m (minimum surface tension of 35 mN/m) for mixed adsorbed PEI/SDS layers in pure water.^10^

With a pH 4 subphase, layer 3 has 80 ± 20% PEI. This observation supports our explanation of the ellipsometry and BAM results above concerning the presence of a rigid film. The ESs present are composed of a layer of surfactant of 20 ± 1 Å, similar to the thickness obtained for ESs in PLL/SDS films,^35^ and the PEI layer beneath (layer 5) is less compact than the one above it (layer 3). There is more PEI in the film at pH 4 than Γ_PEI_/Γ_SDS_ resolved using the low-*Q*_*z*_ implementation of NR. It is unclear to us currently whether this is a result of isotope-specific effects, as are clearly prevalent in systems that exhibit ESs as noted above, or whether the relatively large uncertainties of the Γ_PEI_ values, due to the low scattering length of the monomer units, need greater consideration.

With a pH 10 subphase, layer 3 has 52 ± 16% PEI, indicating a more solvated aqueous region of the surface monolayer in this case. This result is in agreement with those from ellipsometry and BAM, implying the presence of a less rigid and more mobile film. The ESs present below are composed of a layer of surfactant of 22 ± 1 Å, consistent with patches of SDS bilayer or hemimicelles and similar to the results obtained at pH 4. These data require the presence of a diffuse layer or PEI loops to match Γ_PEI_/Γ_SDS_ from the low-*Q*_*z*_ implementation of NR, which is consistent with the physical picture of the same polyelectrolyte bound to a solid support.^56^ From these data, we can infer that it has been possible to relate structural changes in the self-assembly of hyperbranched P/S films to their morphology for the first time.

## Conclusions

The effects of subphase pH on the properties of P/S films containing a weak hyperbranched polyelectrolyte whose charge density varies strongly with pH, spread on water using a new aggregate dissociation mechanism, have been investigated for the first time in the present work. The use of a selection of surface-sensitive techniques, providing information at different time and length scales, has been critical for elucidation of the behavioral properties of the films. The results of electrophoretic mobility, ellipsometry, BAM, and two implementations of NR confirm that there is a strong influence of the subphase pH on the resulting film structures and morphologies.

For subphases of both pH 4 and 10, the general physical pictures of the structures of spread PEI/SDS films upon compression of the surface area are similar, consisting of a PEI/SDS surface monolayer with bound discrete surfactant ESs wrapped in PEI. BAM images and ellipsometry together have revealed that the films are rigid at pH 4 and mobile at pH 10, ellipsometry has revealed that the films at pH 10 are highly heterogeneous due to the presence of embedded aggregates, a physical picture consistent with that resolved in previous works on static PEI/SDS adsorbed layers,^28^ and the two implementation of NR together necessitate a diffuse layer of PEI loops only at pH 10, which is consistent with an observation made about the same polyelectrolyte on a solid support.^56^ The PEI associated with the surface monolayer is denser at pH 4 than pH 10, which may be linked to the rigid versus mobile character of the films at the two respective pH values. The higher amount of PEI in spread films at pH 10 than pH 4 from the low-*Q*_*z*_ implementation of NR is consistent with charge binding from an electrostatic driving force for the association of the components in the spread films, as a lower charge density is consistent with the presence of a higher amount of PEI to bind to the surfactant in the monolayer at pH 10 compared with pH 4. Indeed, a comparison of the surface stoichiometry has allowed a comparison of the estimated polyelectrolyte charge in the bulk versus under confinement at the interface.

In terms of the comparison between the behavior of hyperbranched PEI/SDS films with linear PLL/SDS films studied previously,^35^ their general interfacial structures are similar to ESs bound to a surface monolayer. In both cases, the ESs have a thickness of ∼20 Å, which matches that of a surfactant a bilayer (or hemimicelles). However, the thickness of the polyelectrolyte layer beneath the ESs is much higher for PEI/SDS films than that obtained for the PLL/SDS films, especially at pH 10 where polyelectrolyte loops are observed. We attribute this difference to the hyperbranched structure and partially charged nature of the polyelectrolyte. While the PLL/SDS films exhibit a plateau in the surface pressure during compression, where the onset coincides with the formation of ESs, the surface pressure continues to increase for spread PEI/SDS films after the onset of ES formation. Also, upon spreading, the surface pressure is higher yet the surface excess of surfactant is lower at pH 10 compared with pH 4. These results suggest that ESs with different proportions of surfactant in the surface monolayer form upon spreading, contrary to the behavior of the PLL/SDS films where the ES onset occurs upon compression beyond a discontinuity in the surface pressure.

These findings evidence our fine tuning of the dynamic properties and structures of spread P/S films at the air/water interface. The results obtained in recent years indicate that we are getting closer to the goal of being able to design P/S films based on dynamic properties or desired structure, controlling parameters, such as polyelectrolyte stiffness, charge density of the polyelectrolyte, ionic strength of the subphase, or charge of the aggregates used to create the films. Such films may be highly useful for a range of technological applications, including CO_2_ capture,^40^ low-work function modifiers in electronic devices,^41^ and different biomedical applications, such as gene transfection or drug and protein delivery.^42^

## Supporting Information Available

Comparison of Π–*A* isotherms recorded with SDS and *d*-SDS, electrophoretic mobility and binding isotherms of PEI/SDS solutions at pH 4 and 10, and application of identical 5- and 6-layer models to full-*Q*_*z*_ NR data (PDF)
