# Supplementary material for: Effects of Charge Density on Spread Hyperbranched Polyelectrolyte/Surfactant Films at the Air/Water Interface
Source: Langmuir. 2023 Oct 15;39(42):14869–79. doi: 10.1021/acs.langmuir.3c01514 (PMC10601538; doi:10.1021/acs.langmuir.3c01514)
Supplement: Supplementary file 1 — la3c01514_si_001.pdf [file la3c01514_si_001.pdf]

Effects of Charge Density on Spread Hyperbranched Polyelectrolyte/Surfactant Films at the Air/Water Interface

Javier Carrascosa-Tejedor,[1,2†] Andrea Tummino,[2,3†] Bence Fehér,[4] Attila Kardos,[4,5] Marina Efstratiou,[1] Maximilian W. A. Skoda,[6] Philipp Gutfreund,[2] Armando Maestro,[7,8] M. Jayne Lawrence,[1] Richard A. Campbell[1*] and Imre Varga[4,5*]

1. Division of Pharmacy and Optometry, Faculty of Biology, Medicine and Health, University of Manchester, Oxford Road, Manchester M13 9PT, UK
2. Institut Laue-Langevin, 71 Avenue des Martyrs, CS20156, 38042 Grenoble, France
3. CEA Commissariat à l'Energie Atomique et aux Energies Alternatives, 17 Rue des Martyrs, 38054 Grenoble Cedex 9, France
4. Institute of Chemistry, Eötvös Loránd University, 112, Budapest H-1518, Hungary
5. Department of Chemistry, Faculty of Education, J. Selye University, 945 01 Komárno, Slovakia
6. ISIS Neutron and Muon Source, Rutherford Appleton Laboratory, Harwell Campus, Didcot OX11 0QX, UK
7. Basque Foundation for Science, Plaza Euskadi 5, Bilbao, 48009, Spain
8. Centro de Fısica de Materiales (CSIC, UPV/EHU) - Materials Physics Center MPC, Paseo Manuel de Lardizabal 5, E-20018 San Sebastián, Spain.

† JCT and AT contributed equally to this paper
*corresponding authors: richard.campbell@manchester.ac.uk and varga.imre@ttk.elte.hu

Number of pages = 6

Number of figures = 6

Number of tables = 1

Supporting Information

1. Comparison of Π–A isotherms recorded with SDS and d-SDS

The SDS and d-SDS used for the full-Q_z implementation of NR in the main text were twice recrystallized. The d-SDS used for the low-Q_z implementation of NR was used as received due to sample limitations. Figure S1 shows data recorded on PEI/SDS films on a subphase at (A) pH 4 and (B) pH 10 involving recrystallized SDS (left panels) and used-as-received d-SDS (right panels). While there are small differences in the isotherms, as can be expected due to isotope-specific effects, the general behavior is equivalent, e.g., a higher starting Π at pH 10 than at pH 4.

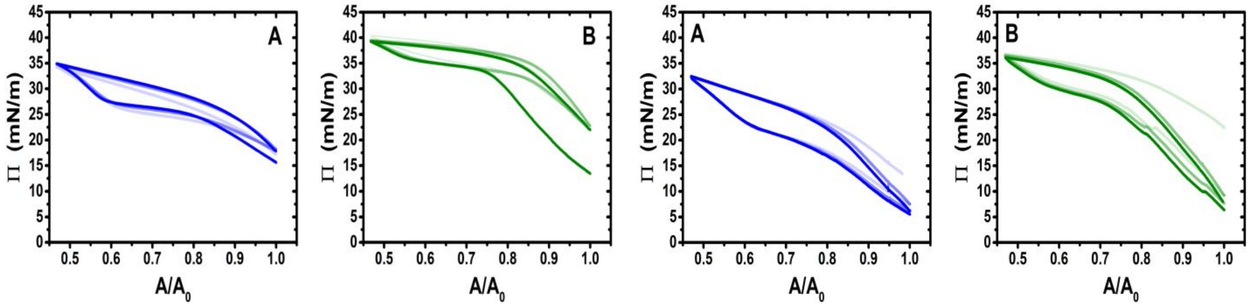

Figure S1. Π–A isotherms of spread PEI/SDS films on a subphase at (A) pH 4 and (B) pH 10 involving recrystallized SDS (left panels) and used-as- received d-SDS (right panels).

2. Electrophoretic mobility and binding isotherms of PEI/SDS solutions at pH 4 and 10

First, we characterize the change in surface charge of PEI/SDS complexes with varying surfactant binding in bulk mixtures, so that a bulk surfactant concentration value can be selected to produce overcharged aggregates for use in the spread film production. We do so at two bulk PEI concentrations, as we go on to calculate the binding isotherm at each pH value.

Figure S2 shows the electrophoretic mobility of PEI/SDS mixtures at (A) pH 4 and (B) pH 10 for 100 and 500 ppm PEI concentrations. As expected, the results show that complexes exhibit positive charge at low bulk surfactant concentrations. In this surfactant concentration range, the samples show much larger positive mobility values at pH 4 than at pH 10, which reflects that the amino groups in PEI are protonated to a much larger extent at low pH. With increasing surfactant concentration, the PEI is first neutralized by the bound surfactant ions, then there is charge reversal at both pH values due to the excess surfactant binding. It may be noted that the scattering intensities at pH 10 for surfactant concentrations much below that of charge neutralization were very low, as shown in Figure S3, so corresponding data in panel B of Figure S2 are omitted as they have low significance and are in fact not needed for the stated objective.

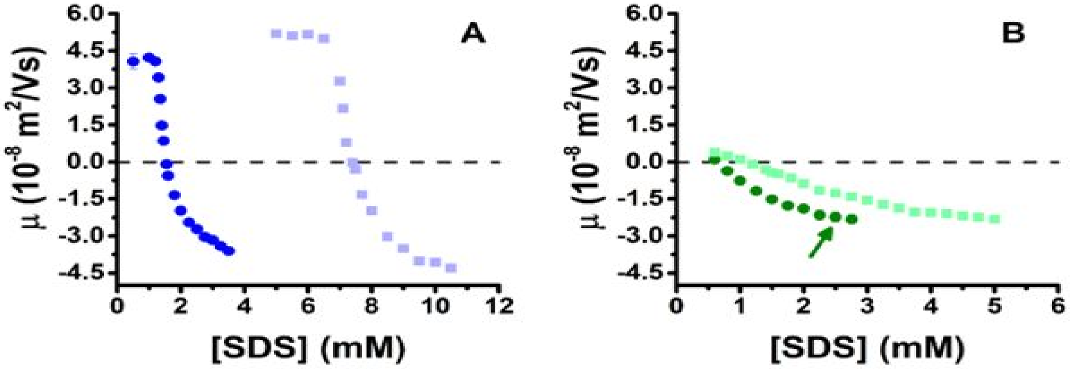

Figure S2. Variation of the electrophoretic mobility of PEI/SDS aggregates with a bulk concentration of SDS using a constant concentration of 100 ppm PEI at (A) pH 4 and (B) pH 10. Two concentrations of PEI were used: 100 ppm (dark circles) and 500 ppm (light squares). The black dashed lines indicate neutral charge. The arrow in panel B indicates the composition used to produce aggregates for spread film preparation on subphases at both pH values.

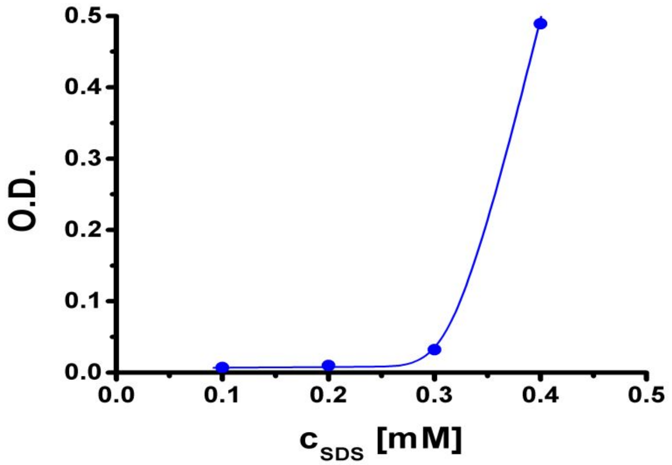

Figure S3. Optical density of PEI/SDS solutions in the range of 0.1–0.4 mM SDS concentration at pH 10.

The binding isotherm, β, which is the ratio of the concentration of bound surfactant (c_{Bound}) to the polyelectrolyte concentration expressed per monomer unit (c_P), can be plotted as a function of equilibrium free surfactant concentration (c_{eq}). To generate a binding isotherm plot for PEI/SDS mixtures at each pH value, we used the electrophoretic mobility method that was proposed by Mészáros et al. (*Langmuir* **2006**, *22* (17), 7148–7151). Namely, since it is accepted that P/S complexes that contain the same amount of bound surfactant have equivalent electrophoretic mobilities regardless of the polyelectrolyte concentration, variation of β with respect to c_{eq} is calculated for any electrophoretic mobility value in the surfactant concentration range where the electrophoretic mobility of the samples varies significantly at two polyelectrolyte concentrations. This is achieved by resolving the total surfactant concentrations ($c_0^{(1)}$, $c_0^{(2)}$) where the complexes have a given electrophoretic mobility at two different polyelectrolyte concentrations ($c_P^{(1)}$, $c_P^{(2)}$),

$$c_0^{(1)} = c_{eq} + c_P^{(1)}\beta \quad \text{(S1)}$$

$$c_0^{(2)} = c_{eq} + c_P^{(2)}\beta \quad \text{(S2)}$$

The resulting data are shown in Figure S4. The level of surfactant binding is much higher at pH 4, but it may be noted that even in this case only ~ 60% of the polymer repeat units are charged at stoichiometric surfactant binding. This amount increases only to a limited extent even when the free surfactant concentration increases by an order of magnitude. Interestingly, the equilibrium free surfactant concentration where charge neutralization takes place is much lower at pH 4 (0.1 mM SDS compared to 0.5 mM SDS at pH 10), which implies that the driving force of surfactant binding is much larger at low pH due to the higher charge density of the polymer. At pH 10, only ~ 5% of the PEI repeat units are charged at stoichiometric surfactant binding. However, this low level of surfactant binding increases significantly with increasing bulk surfactant concentration. Our results paint a qualitatively consistent picture with previous binding data on the PEI/SDS mixed system by Mészáros (*Langmuir* **2006**, *22* (17), 7148–7151).

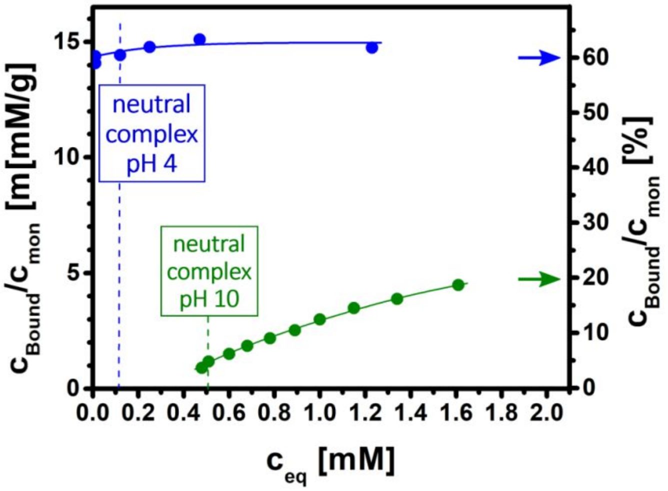

<u>Figure S4.</u> Binding isotherm of SDS on PEI at pH 4 (blue) and pH 10 (green). Lines through the data points are guides to the eye. The left axis has the binding isotherm with c_{mon} expressed per mass of each monomer unit while the right axis is expressed per monomer unit. The dashed vertical lines indicate the concentration at which neutral PEI/SDS aggregates appear, and extrapolations of the corresponding surfactant binding at 2.5 mM are indicated with arrows.

These results highlight the impact of pH on PEI/SDS interactions, which was part of our underlying hypothesis that they can greatly affect the properties of PEI/SDS spread films. To emphasize effects on the film properties from the subphase pH, as opposed to different films formed from aggregates prepared at different pH values, aggregates were prepared at pH 10, the natural pH of mixing the components (*Langmuir* **2003**, *19* (3), 609–615), for spreading on subphases at pH 4 and 10. A total SDS concentration of 2.5 mM was chosen to produce overcharged aggregates with 100 ppm PEI at its natural pH value (green arrow in panel B of figure SI1) for film creation on subphases of both pH values. At the chosen surfactant concentration, an estimate of 60% binding at pH 4 (blue arrow in Figure SI2) and 20% binding at pH 10 (green arrow in Figure S2) were used in the calculation of the scattering length of PEI in the NR data analysis (Table 1).

3. Application of identical 5- and 6-layer models to full-Q_z NR data

As mentioned in the main text, data from the d-SDS/D_2O contrast do not fit to a global fit of the same model as the other two measured contrasts unless the volume fraction of the ESs is varied. Figure S5 shows a comparison between the fit of data from the d-SDS/D_2O contrast for compressed PEI/SDS spread films presented in the main text, corresponding to a PEI/SDS monolayer, and a simulation of data of the same contrast considering the parameters obtained for the 5-layer fit that include ESs in the co-refinement of the data from the d-SDS/ACMW and hSDS/D_2O contrasts. The ESs that results in a satisfactory fit for these other first two contrasts clearly do not represent the structure of the film of the sample with d-SDS/D_2O for either pH value. This result is in keeping with other recent studies discussed in the main text.

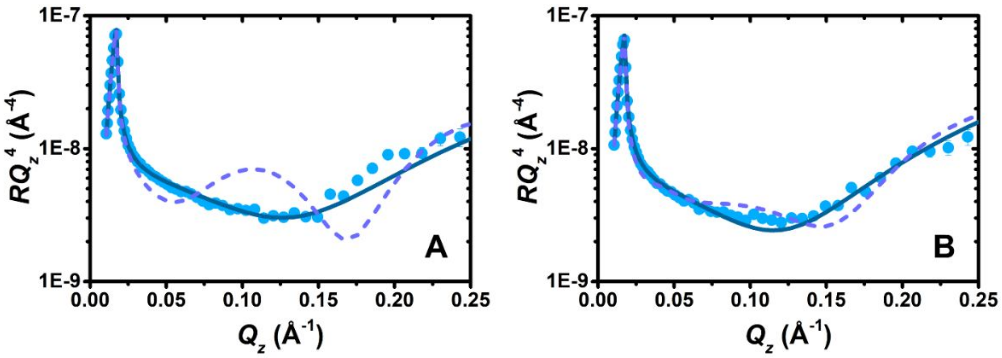

Figure S5. Neutron reflectivity profiles and fits of compressed PEI/SDS spread films in the d-SDS/D_2O contrast at (A) pH 4 and (B) pH 10. The data are represented by the points while the solid and dashed lines correspond to the fits in the manuscript (PEI/SDS monolayer) and a simulation of the model obtained in the co-refinement of d-SDS/ACMW and hSDS/D_2O (PEI/SDS monolayer with ESs), respectively.

Tables S1 show the fitting parameters for the measurements at pH 4 and pH 10, respectively, while Figure S6 shows the reflectivity and volume fraction profiles obtained by co-refinement of the three contrasts using 5 or 6 layers for all of them. The model fits to data only from the d-SDS/ACMW and SDS/D_2O contrasts deviate from the reflectivity profiles with respect to those presented in the main manuscript, and the fits are particularly poor for data from the d-SDS/D_2O contrast. While the χ^2 of the fits without including this contrast is 6.9 and 7.9 for pH 4 and 10, respectively, these values increase to 19.8 and 21.1 when it is included. These results further evidence the need to vary the coverage of ESs according to the isotopic contrast.

Table S1. Thickness (d_i), scattering length density (ρ_i), and composition obtained from the three-contrast fits of the PEI/SDS films spread on a subphase adjusted to pH 4 and 10, where *i* is the layer number.

Layer	Parameter	pH 4	pH 10
1	d_1 (Å)	9	9
	Composition (*V*%)	100% SDS chains	100% SDS chains
2	d_2 (Å)	4	4
	Composition (*V*%)	39% SDS heads<br>50% PEI<br>11% solvent	39% SDS heads<br>50% PEI<br>11% solvent
3	d_3 (Å)	5	6
	Composition (*V*%)	55% PEI<br>45% solvent	50% PEI<br>50% solvent
**4 (*excluding dSDS/D_2O contrast)**	d_4 (Å)	23	22
	Composition (*V*%)*	40% SDS<br>60% solvent	38% SDS<br>62% solvent
**5 (*excluding dSDS/D_2O contrast)**	d_5 (Å)	8	6
	Composition (*V*%)	22% PEI<br>78% solvent	22% PEI<br>78% solvent
**6 (*excluding dSDS/D_2O contrast)**	d_6 (Å)	-	100
	Composition (*V*%)	-	5% PEI<br>95% solvent

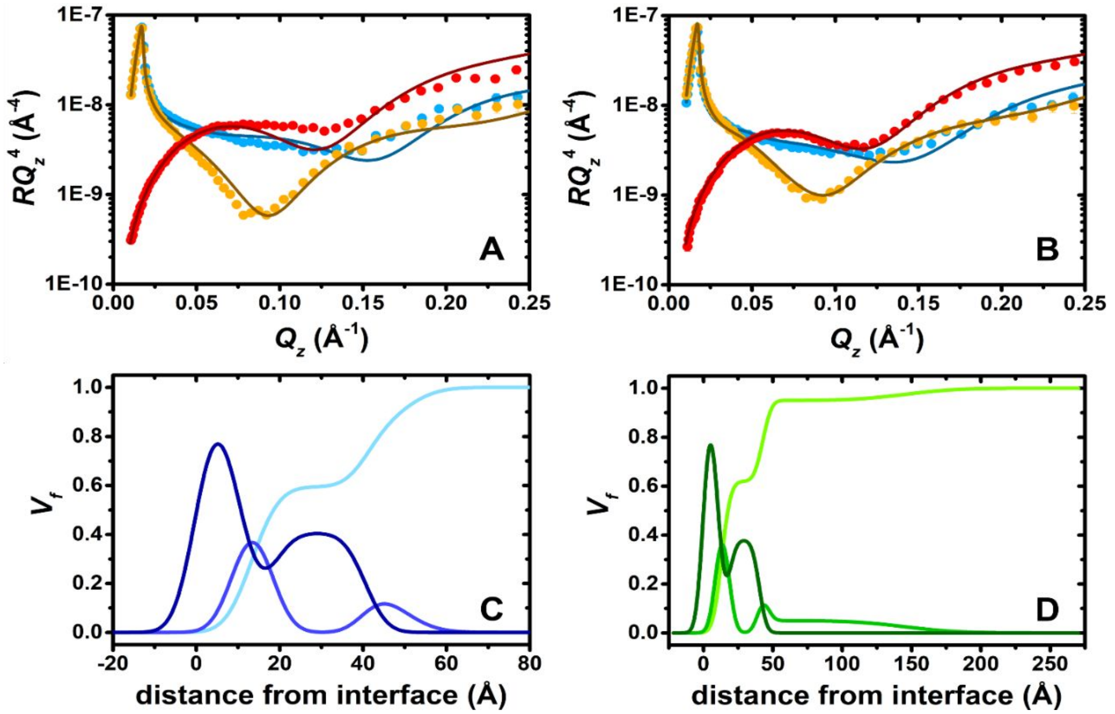

Figure S6. Neutron reflectivity (A, B) data (points) and model fits (lines) of spread PEI/SDS films in three isotopic contrasts involving PEI with d-SDS in ACMW (red) and (blue) SDS in D_2O (orange) of spread PEI/SDS films on pure water subphases adjusted to (A, C) pH 4 and (B, D) pH 10 and then held at 40 mN/m, resolved using the full-Q_z implementation of NR; (C, D) resulting volume fraction profiles where surfactant is dark blue/dark green, polyelectrolyte is blue/green and solvent is light blue/light green, respectively.
